# 

**Published:** 1978

**Authors:** 


### Contents

3 Porphyrias and Mental Handicap
J. Jancar

7 Associations between HLA Antigens and Disease
Geoffrey H. Tovey

